# Identification and Function of Myeloid Differentiation Factor 88 (MyD88) in *Litopenaeus vannamei*

**DOI:** 10.1371/journal.pone.0047038

**Published:** 2012-10-12

**Authors:** Shuang Zhang, Chao-Zheng Li, Hui Yan, Wei Qiu, Yong-Gui Chen, Pei-Hui Wang, Shao-Ping Weng, Jian-Guo He

**Affiliations:** 1 MOE Key Laboratory of Aquatic Product Safety/State Key Laboratory of Biocontrol, School of Life Sciences, Sun Yat-sen University, Guangzhou, People's Republic of China; 2 School of Marine Sciences, Sun Yat-sen University, Guangzhou, People's Republic of China; National Institute on Aging, United States of America

## Abstract

Myeloid differentiation factor 88 (MyD88) is a universal and essential signaling protein in Toll-like receptor/interleukin-1 receptor-induced activation of nuclear factor-kappa B. In this study, two MyD88 protein variants (LvMyD88 and LvMyD88-1) were identified in *Litopenaeus vannamei*. The LvMyD88 cDNA is 1,848 bp in length and contains an open reading frame (ORF) of 1,428 bp, whereas the LvMyD88-1 cDNA is 1,719 bp in length and has an ORF of 1,299 bp. Both variants encode proteins with death and Toll/interleukin-1 receptor domains and share 91% sequence identity. In healthy *L. vannamei*, the LvMyD88 genes were highly expressed in hemocytes but at a low level in the hepatopancreas. The LvMyD88s expression was induced in hemocytes after challenge with lipopolysaccharide, CpG-ODN2006, *Vibrio parahaemolyticus*, *Staphyloccocus aureus*, and white spot syndrome virus, but not by poly I∶C. Overexpression of LvMyD88 and LvMyD88-1 in *Drosophila* Schneider 2 cells led to activation of antimicrobial peptide genes and wsv069 (ie1), wsv303, and wsv371. These results suggested that LvMyD88 may play a role in antibacterial and antiviral response in *L. vannamei*. To our knowledge, this is the first report on MyD88 in shrimp and a variant of MyD88 gene in invertebrates.

## Introduction

Innate immunity is the first-line of host defense in multicellular organisms against infectious oppathogens [1] and is activated upon recognition of microbial derived pathogen-associated molecular patterns (PAMPs) by host pattern recognition receptors (PRRs) [2]. Toll-like receptors (TLRs), an important family of the PRRs which are well characterized in vertebrates, initiate a signaling cascade which leads to activation of the Myeloid differentiation factor 88 (MyD88) and the transcription factor nuclear factor-kappaB (NF-κB) [3]–[5]. MyD88 consists of a Toll/interleukin-1 receptor (TIR) domain and a death domain and is the common signaling adaptor protein shared by all TLRs except TLR3 [6]–[8]. The TIR domain is essential to the interactions between TLRs and MyD88. The death domain, in turn, associates with the death domain of interleukin-1 receptor associated kinase (IRAK), to trigger downstream signaling cascades that lead to the activation of the NF-κB [9]–[11].

MyD88 was first identified in 1990 [12] and has been extensively studied in many species, including human [13], porcine [14], mouse [15], chicken [16], reptiles [17], [18], fish [19]–[26], scallop [27] and flies [28]. To date, variants of MyD88 have been reported only in several vertebrates such as humans [29], mice [30] and chicken [18]. Recent studies have shown that MyD88 genes are duplicated in common carp [25]. Although the MyD88 gene has been sequenced in diplostraca and anostraca [31] little is known about its extence in in penaeidae.

The Pacific white shrimp, *Litopenaeus vannamei*, is distributed along the Pacific coast ranging from the Gulf of California to the northern Peru and has become one of the most important economic penaeid shrimps worldwide, particularly in the Eastern Pacific region and Asia [32]. With rapid expansion of farming, bacterial and viral diseases have become a major concern, causing substantial economic losses in many countries since the 1990s [33]–[35]. For example, the white spot syndrome virus (WSSV) is one of the most common and destructive pathogen and is able to cause 100% mortality within 3 days to 10 days after infection [36]. Study on the *L. vannamei* immune system is much needed in order to design better strategies for disease prevention and control.

Our previous studies suggest that a signaling cascade similar to the TLR/MyD88/Tube/Pelle/TRAF6/NF-κB pathway may exist in *L. vannamei*, and could be activated by wsv449, to upregulate the expression of wsv069 (ie1), wsv303, and wsv371 [37]. Several key genes in this pathway, including the Toll genes, the Pelle gene, the TRAF6 gene, and the Rel/NF-κB homologous genes (Dorsal and Relish), have been characterized [37]–[42]. In this study, two variants of the MyD88 gene were identified in *L. vannamei* and their function was studied in signal transduction in response to different stimuli.

## Materials and Methods

### Microorganisms

Gram-negative *Vibrio parahaemolyticus* were cultured in a thiosulfate-citrate-bile salts-sucrose (TCBS) agar culture medium at 30°C for 18 h. Gram-positive *Staphyloccocus. aureus* were cultured in a nutrient broth agar at 37°C for 24 h. The *V. parahaemolyticus* and *S. aureus* cells were centrifuged at 5000 g for 10 min at 4°C, washed with 1×PBS (8 g NaCl, 0.2 g KCl, 1.44 g Na_2_HPO_4_, and 0.24 g K_2_HPO_4_, diluted with dH_2_O to 1 litre and with the pH adjusted to 7.3), and then resuspended in 1×PBS. The bacterial concentration was quantified as the microbial colony-forming units per milliliter (CFU/ml) and the bacterial solution adjusted to 10^6^ CFU/ml.

The WSSV-infected *L. vannamei* were collected from the Hengxing shrimp farm in Zhanjiang, Guangdong Province, China, and stored at −80°C. Muscle samples (0.1 g) from the WSSV-infected *L. vannamei* were homogenized in 1 ml of 1×PBS and centrifuged at 5000 g for 15 min at 4°C. The supernatant was filtered through a 0.45 µm membrane, and used as the WSSV inocula.

### Total RNA isolation, cDNA synthesis, and genomic DNA preparation

*L. vannamei* (∼8 g to 10 g each) was purchased from a shrimp market in Guangzhou, Guangdong Province, China. The RNeasy Mini Kit (Qiagen, Germany) was used to extract the total RNA from tissue samples. Residual genomic DNA was removed by RNase-free DNase I (Qiagen, Germany). The total RNA was then reverse-transcribed into first strand cDNA using a PrimeScript™ 1st Strand cDNA Synthesis Kit (TaKaRa, China) for gene cloning. For real-time quantitative polymerase chain reaction (qPCR) analysis, the cDNA samples were prepared using the PrimeScript™ RT reagent kit (TaKaRa, China). The cDNA template for the rapid amplification of the cDNA ends (RACE) PCR was prepared using the SMARTer™ RACE cDNA amplification kit (Clontech, USA). Genomic DNA was extracted from muscle samples using the Universal Genomic DNA Extraction Kit (TaKaRa, China).

### Cloning the cDNA and genome of LvMyD88

Degenerate primers for cloning of LvMyD88, DPMyD88F and DPMyD88R (Table 1), were designed from conserved regions of the published MyD88 nucleotide sequences of *Tribolium castaneum* (EFA01304), *Drosophila melanogaster* (NP_610479), *Mus musculus* (NP_034981) and *Homo sapiens* (AAB49967). A cDNA fragment of LvMyD88 was initially amplified by PCR with degenerate primers using hemocytes derived cDNA. Based on the cDNA fragment, the full-length MyD88 cDNA was obtained via the 5′ and 3′RACE PCR as described previously [42]. Briefly, 5′ RACE1 and 3′ RACE1 primers (Table 1) were used for the first round 5′-end and 3′-end RACE-PCR,respectively, using the following program: 94°C for 3 min, 10 cycles of 94°C for 20 s, 62°C for 30 s (a decrease of 0.5°C per cycle), 72°C for 2 min, 30 cycles of 94°C for 20 s, 57°C for 30 s, 72°C for 2 min, and a final extension at 72°C for 10 min. These PCR conditions were also applied to the second-round 5′-end and 3′-end RACE PCR where 5′ RACE2 and 3′ RACE2 primers were used respectively. The genomic DNA sequences of LvMyD88 were obtained by PCR using the genomic DNA (the primers are listed in Table 1) using the following program: 94°C for 3 min, 34 cycles of 94°C for 30 s, 57°C for 30 s, 72°C for 3 min, followed by a final extension at 72°C for 10 min. The PCR products were cloned into the pMD-20 vector (Takara, Japan) and sequenced. The gene sequences obtained in this study have been deposited in the NCBI GenBank (http://www.ncbi.nlm.nih.gov/genbank/).

**Table 1:** PCR primers.

Primers	Primer sequences (5′-3′)
**For cDNA cloning**	
DPMyD88F^a^	CTGTACGCCCACGMNGAYA
DPMyD88R^a^	ADTADGGGACGTAGATGTTCTTG
5′ RACE1	CACCGCTCCATGATGAGTTTAAC
5′ RACE2	CACCAATAAGGTCTCTGTTCTTGTG
3′ RACE1	CTGCTGTGATAAGTTTCTGCCA
3′ RACE2	TAGGGCAAAGGGCTATTGGAAC
**For Genomic DNA cloning**	
GLvMyD88-F1	TCGGGAAGAAGTGGCAGAG
GLvMyD88-R1	CTACAGTAAGAATTTGGCTATCTT
GLvMyD88-F2	TTGACCAGAGGGTGCCACAGGTAG
GLvMyD88-R2	CCGCTCCATGATGAGTTTAACA
GLvMyD88-F3	GGCAACCACAAAATACTC
GLvMyD88-R3	CAACCTTAGTATATAGATGCTCCAGTC
GLvMyD88-F4	ATGAATTGTGATAAATTTGC
GLvMyD88-R4	GGAAAACCCTGCATTGCC
**For Protein expression**	
pAcLvMyD88-F^b^	CCG**CTCGAG**ATGTCATTTCGTCGGGAAGA
pAcLvMyD88-R^b^	CGG**GGGCCC**CCCAGGAATTTTGATTTTTTTC
**For qPCR**	
LvMyD88-F1	GCTGTTCCACCGCCATTT
LvMyD88-R1	GCATCATAGTGCTGTAGTCCAAGA
LvMyD88-F2	GGCAAAGGGCTATTGGAACTAT
LvMyD88-R2	ATGATCCAGACACCTCTCGTATTC
LvEF1α-F1	AAGGCCCTCAAGAAGAAGTAAAT
LvEF1α-R1	TTGACAACCATACCTGGCTTC
LvEF1α-F2	TGCACCACGAAGCCCTTAC
LvEF1α-R2	CAGGGTGGTTGAGGACGATC

a M = A or C; N = A, C, G or T/U; Y = C or T; D = not C.

b Nucleotides in bold represent the restriction sites introduced for cloning.

### Bioinformatics analysis

The BLAST program (http://www.ncbi.nlm.nih.gov/BLAST/) was used to analyze the nucleotide sequences and to search for protein sequences in the databases. Multiple sequence alignment was generated using the ClustalX 2.0 program (http://www.ebi.ac.uk/tools/clustalw2). The simple modular architecture research tool (SMART, http://smart.embl-heidelberg.de) was used to analyze the protein domain topology. The neighbor-joining phylogenic trees were constructed based on the amino acid sequences using the MEGA 4.0 software (http://www.megasoftware.net/index.html) and bootstrapped for 1000 times.

### Immune challenge and gene expression analysis

Twelve tissues, including the hemocytes, hepatopancreas, gill, heart, stomach, pyloric caecum, nerve, epithelium, eyestalk, intestine, seminal vesicle and muscle, were obtained from healthy *L. vannamei* for RNA extraction. For the reason that there were no specific primers to detect the expression level of LvMyD88-1, the expression level of LvMyD88 and LvMyD88s (the amount of LvMyD88 and LvMyD88-1) were investigated using primers LvMyD88-F1/LvMyD88-R1 and LvMyD88-F2/LvMyD88-R2, respectively. On the basis, the expression level of LvMyD88-1 was calculated using the method put forward by Pfaffl [43]. The PCR was performed in a LightCycler (Roche) with the following program: one cycle at 95°C for 30 s, 40 cycles of 95°C for 5 s, 57°C for 30 s, and 78°C for 5 s. Three replicate qPCRs were performed per sample. Elongation factor 1α (EF1α) was used as the internal control.

For the challenge experiments, healthy *L. vannamei* was intramuscularly injected with LPS (2 µg/g), poly I∶C (2 µg/g), CpG-ODN2006 (2 µg/g), *V. parahaemolyticus* (5.5×10^6^ CFU/g), *S. aureus* (2.5×10^6^ CFU/g), or WSSV (10^6^ copies/g) at the third abdominal segment. *L. vannamei* injected with PBS were used as controls. At 0, 4, 8, 12, 24, 36, 48, and 72 h post-injection, three animals from each group were randomly sampled for hemocyte collection. The relative mRNA expression of the LvMyD88 genes was detected by qPCR use the same program described above.

### Plasmid construction

For protein expression in *Drosophila* Schneider 2 (S2) cells, pAc5.1/V5-His A (Invitrogen, USA) and the PCR products amplified with pAcLvMyD88F and pAcLvMyD88R were digested with resitriction enzymes Kpn I and Apa I (Takara, Japan) and purified. The mixture was ligated at 4°C overnight and then transformed into the DH5α competent cells. Positive clones were confirmed by colony PCR and sequenced. In our previous studies [37]–[42], several luciferase reporter vectors were constructed using the promoter sequences of following genes: the *Drosophila* AMPs, Attacin A (AttA), Drosomycin (Drs), and Metchnikowin (Mtk); the *L. vannamei* AMP Penaeidin4 (PEN4); the *Penaeus monodon* AMP Penaeidin (PEN309, PEN453, and PEN536); and wsv069 (ie1), wsv303, and wsv371. Luciferase reporter genes, including pGL3-AttA, pGL3-Drs, pGL3-Mtk, pGL3-PEN4, pGL3-PEN309, pGL3-PEN453, pGL3-PEN536, pGL3-wsv069, pGL3-wsv303, and pGL3-wsv371, have been shown to be regulated through NF-κB activation [37], [39], [40], [44]–[46].

### Dual-luciferase reporter assay

Given the unavailability of a permanent shrimp cell line, *Drosophila* S2 cells (Invitrogen, USA) were used to perform the functional studies of LvMyD88. The S2 cells were maintained at 28°C overnight in a *Drosophila* serum-free medium (SDM; Invitrogen, USA) supplemented with 10% fetal bovine serum (FBS) prior to DNA transfection. The plasmids were then transfected with the Cellfectin II reagent (Invitrogen, USA) according to the manufacturer's instructions. For the dual-luciferase reporter assays, the S2 cells cultured in 96-well plates (TPP, Switzerland) were transfected using 0.3 µg expression plasmids, 0.2 µg reporter gene plasmids, and 0.02 µg pRL-TK renilla luciferase plasmid (Promega, USA). The pRL-TK renilla luciferase plasmid was transfected alone as an internal control. Firefly and renilla luciferase activities were measured using a Dual-Luciferase Reporter Assay System (Promega, USA) according to the manufacturer's instructions. All assays were performed in three independent transfections.

### Statistical analysis

The student's t-test was used to compare the means of two samples using Microsoft Excel wherever applicable. In all cases, differences were considered significant at *p*<0.05. All experiments were repeated at least three times. The data are presented as the mean ± standard error (standard error of the mean, SEM).

## Results

### cDNA cloning and bioinformatics analysis of LvMyD88

**Figure 1:**
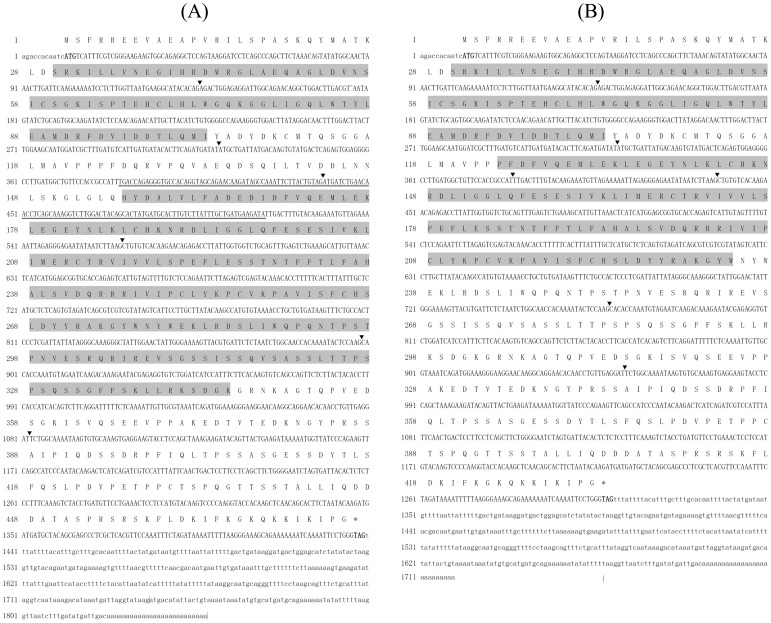
Nucleotide and deduced amino acid sequences of (A) LvMyD88 and (B) LvMyD88-1 from *Litopenaeus vannamei*. The nucleotide (lower row) and deduced amino acid (upper row) sequences are shown and numbered on the left. The translation initiation codon (ATG) and stop codon (TAA or TGA) are in bold. The N-terminal death domain (DD, residues 30–103) and C-terminal Toll/interleukin-1 receptor (TIR) domains (residues 156–345 and 124–234 in LvMyD88 and LvMyD88-1, respectively) are shaded. The nucleotides that are absent in LvMyD88-1 were underlined. ▾ denote the exon-exon boundaries.

**Figure 2:**
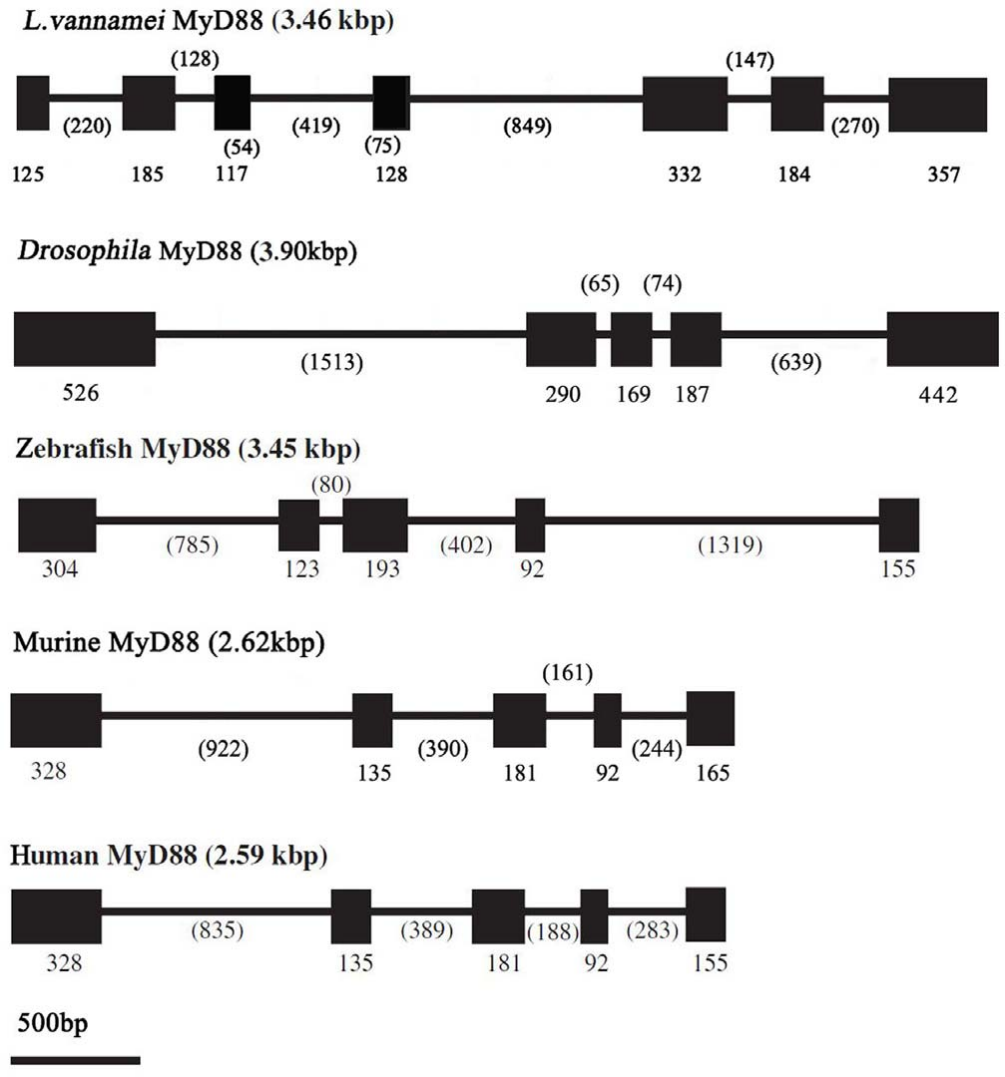
Comparison of the MyD88 gene structures in *L. vannamei*, Drosophila, zebrafish, chicken, mouse, and human. Boxes in represent exons, and the numbers indicate the length (bp) of the exons and introns.

Two LvMyD88 variants, namely, LvMyD88 and LvMyD88-1, were found in *L. vannamei*. The full length cDNA of LvMyD88 comprises 1,848 bp with an ORF of 1,428 bp, an 11 bp 5′ untranslated region, and a 409 bp 3′ untranslated region (Fig. 1A). The full length cDNA of LvMyD88-1 contains 1,719 bp with an ORF of 1,299 bp, an 11 bp 5′ untranslated region, and a 409 bp untranslated region (Fig. 1B). The deduced amino acid sequence of LvMyD88-1 displays 91% identity with LvMyD88. The genomic sequence of LvMyD88 was also obtained, exhibiting a genomic organization which is different to that of the MyD88 genes from *Drosophila*, zebrafish, chicken, mouse, and human. The LvMyD88 genes consist of 7 exons and 6 introns whilst other known MyD88 genes have 5 exons and 4 introns (Fig. 2). Analysis of genome sequences showed that all the exon-intron boundaries in LvMyD88 conform to the consensus GT/AG rule for splicing [47]. However, neither splice donor consensus (GT) nor splice acceptor consensus (AG) was found in the sequences missing in LvMyD88-1. The sequences were deposited in the NCBI GenBank under Accession No. JX073566, No. JX073567 and No. JX073568.

**Figure 3:**
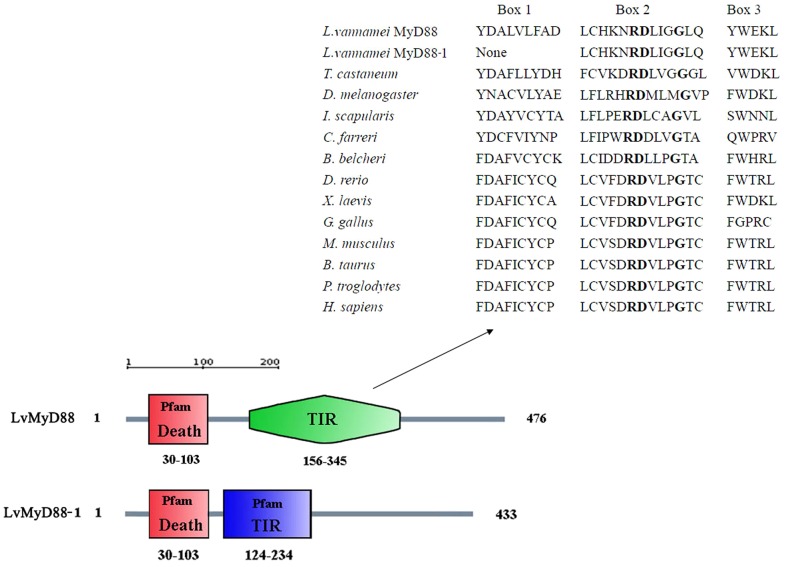
Schematic description of the domain topologies of LvMyD88 and LvMyD88-1. LvMyD88 and LvMyD88-1 contain the same N-terminal Death domains (residues 30–103) and different C-terminal TIR domains (residues 156–345 and 124–234, respectively). The TIR domains from *L. vannamei* with other species were shown.

To determine the structural domains of LvMyD88, the amino acid sequence was analyzed using the SMART program.LvMyd88 and LvMyD88-1 possess an identical death domain at the N terminus but different C-terminal TIR domains. Three highly conserved regions (Box1, Box2, and Box3) present in most TIR domains were found in LvMyD88. However, the Box1 region was absent in LvMyD88-1. The RDXΦ1Φ2G motif where X represents any amino acid and Φ represents a hydrophobic residue is known to be essential for the interaction of TLRs with MyD88 [48]. This motif can be found in the Box2 of LvMyD88 and LvMyD88-1 (Fig. 3).

**Figure 4:**
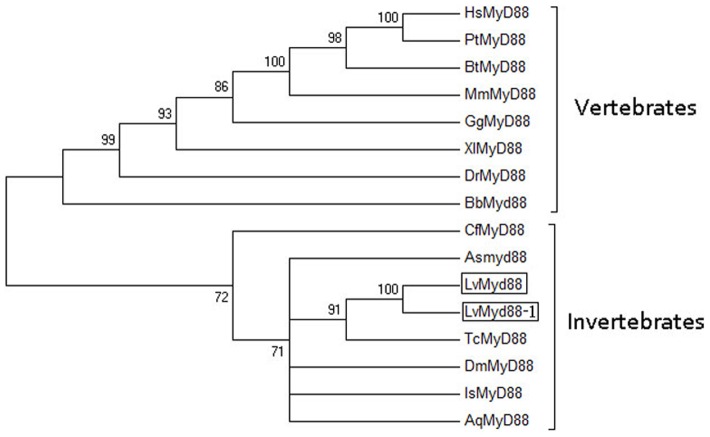
Phylogenetic tree analysis. A rooted tree was constructed via the neighbor-joining method and bootstrapped 1000 times using the MEGA 4.0 software (http://www.megasoftware.net/index.html). LvMyD88 is boxed. LvMyD88, *L. vannamei* MyD88 (Accession No. JX073566); LvMyD88-1, *L. vannamei* MyD88-1 (Accession No. JX073567); AqMyD88, *Amphimedon queenslandica* MyD88 (Accession No. ADR78337); AsMyD88, *Artemia sinica* MyD88 (Accession No. AEJ08192); BbMyD88, *Branchiostoma belcheri* MyD88 (Accession No. ABQ32299); BtMyD88, *Bos taurus* MyD88 (Accession No. DAA17130); CfMyD88, *Chlamys farreri* MyD88 (Accession No. ABB76627); DmMyD88, *Drosophila melanogaster* MyD88 (Accession No. AAF58953); DrMyD88, *Danio rerio* MyD88 (Accession No. NP_997979.2); GgMyD88, *Gallus gallus* MyD88 (Accession No. NP_001026133); HsMyD88, *Homo sapiens* MyD88 (Accession No. AAC50954); IsMyD88, *Ixodes scapularis* MyD88 (Accession No. XP_002407372); MmMyD88, *Mus musculus* MyD88 (Accession No. AAC53013); PtMyD88, *Pan troglodytes* MyD88 (Accession No. NP_001123935); TcMyD88, *Tribolium castaneum* MyD88 (Accession No. EFA01304); and XlMyD88, *Xenopus laevis* MyD88 (Accession No. NP_001081001).

The NCBI BLAST search tool and pairwise ClustalW2 program were used to calculate the percentage of amino acid identity among MyD88s. The LvMyD88s had highest sequence identity (80%) with *Xenopus laevis* MyD88 (Table 2), and 50%, 31% and 43% sequence identity with the MyD88s from three invertebrate species, *T. castaneum*, *D. melanogaster* and *Ixodes scapularis*, respectively. In addition, a phylogenetic tree was constructed to determine the evolutionary relationship of LvMyD88 with other known MyD88 family members, showing that LvMyD88 belonged to the invertebrate group and was closely related to *T. castaneum* MyD88 (Fig. 4).

**Table 2:** Full-length amino acid sequence.

Species	Accession number	Amina acids	Identity %		
			Full-length	DD	TIR
*Tribolium castaneum*	EFA01304	400	50%	41%	42%
*Drosophila melanogaster*	AAF58953	537	31%	43%	35%
*Ixodes scapularis*	XP_002407372	364	43%	39%	28%
*Chlamys farreri*	ABB76627	367	40%	36%	39%
*Branchiostoma belcheri*	ABQ32299	295	42%	34%	38%
*Xenopus laevis*	NP_001081001	283	80%	32%	36%
*Danio rerio*	NP_997979.2	284	30%	33%	35%
*Gallus gallus*	NP_001026133	376	33%	34%	38%
*Mus musculus*	AAC53013	296	37%	29%	36%
*Bos taurus*	DAA17130	296	30%	28%	37%
*Pan troglodytes*	NP_001123935	296	31%	30%	36%
*Homo sapiens*	AAC50954	296	31%	30%	36%

Death domain (DD) and Toll/interleukin-1 receptor (TIR) domain identities of LvMyD88 with other species.

### Tissue distribution of LvMyD88s in healthy *L. vannamei*

**Figure 5:**
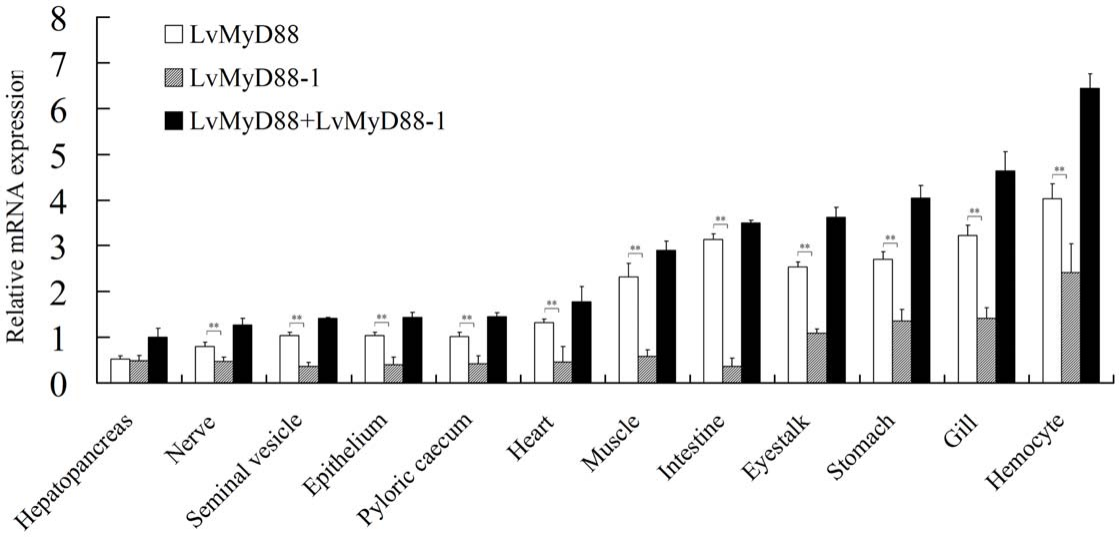
Tissue distribution of LvMyD88 expression in healthy *L. vannamei*. Ten animals were used for tissue sampling. LvEF1α was used as the internal control to normalize the cDNA template used for real time PCR analysis. The data are presented as the mean ± standard error (standard error of the mean, SEM) and *p*<0.05 is considered significantly different.

The constitutive expression of LvMyD88, LvMyD88-1 and LvMyD88s (the amount of LvMyD88 and LvMyD88-1) were confirmed by RT-PCR in all the examined tissues, including hepatopancreas, gill, muscle, intestine, pyloric caecum, epidermis, nerve, heart, stomach, eyestalk, seminal vesicle and hemocytes. LvMyD88s were expressed highest in hemocytes and lowest in hepatopancreas. In general, the LvMyD88 expression level was higher than that of LvMyD88-1 in all tissues examined (Fig. 5).

### LvMyD88 expression in immune-challenged *L. vannamei*

Peptidoglycan (PGN) and lipoteichoic acid (LTA) in gram-positive bacteria can be recognized by TLR2 [10]. Poly I∶C, LPS and CpG-ODN2006 are the ligands for TLR3, TLR4 and TLR9, respectively [11]. In this study, gram-positive bacteria *S. aureus*, poly I∶C, LPS and CpG-ODN2006 were chosen to challenge the *L. vannamei*. In addition, gram-negative pathogenic bacterium *V. parahaemolyticus* and one of the most common and most destructive viral pathogens (WSSV) in shrimp aquaculture were also used to for the challenge experiments [36]. Considering that LvMyD88s (the amount of LvMyD88 and LvMyD88-1) were expressed highly in hemocytes, we selected hemocytes to study LvMyD88s expression in response to immune challenges.

**Figure 6:**
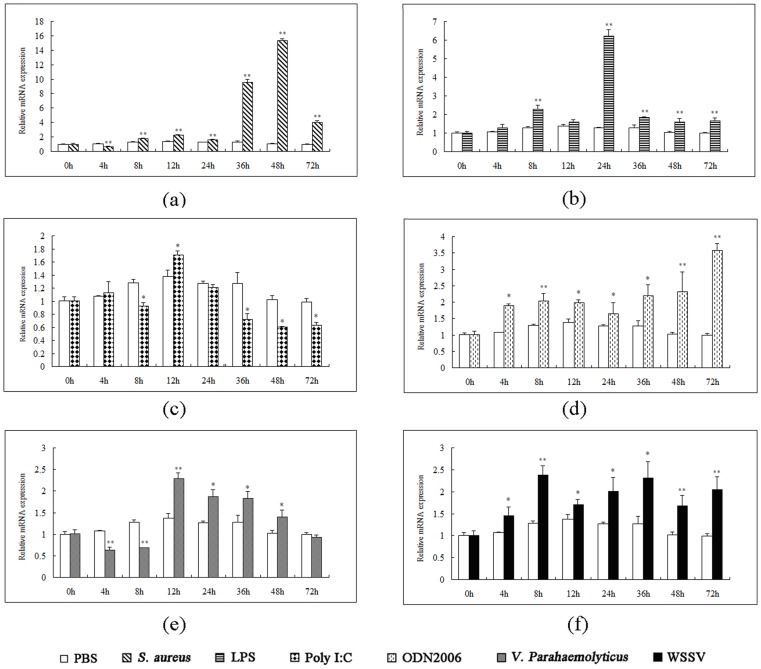
Temporal expression of LvMyD88 in immune-challenged *L. vannamei*. The relative expression level of target genes was normalized to LvEF1a. The relative expression of LvMyD88 in treated groups [(a) *Staphyloccocus aureus*, (b) lipopolysaccharide (LPS), (c) poly I∶C, (d) CpG-ODN2006, (e) *Vibrio parahaemolyticus*and, (f) white spot syndrome virus (WSSV)] were compared with the control group. The results were based on three independent experiments and expressed as mean values ± SD. The statistical significance was calculated using Student's *t*-test (* indicates *p*<0.05 and ** indicates *p*<0.01 compared with control).

*S. aureus* challenge increased LvMyD88s transcripts. The LvMyD88s expression level reached its peak at 48 h post-injection and sharply increase to approx 15.3 times higher than that of the control [Fig. 6(a)]. After the LPS challenge, the level of LvMyD88 transcripts started to increase at 4 h and reached the peak at 24 h, with approx 5- folds increase compared with control [Fig. 6(b)]. In contrast, injection of poly I∶C reduced gene expression at 8 h whilst induced gene expression at 12 h. Subsequently, the LvMyD88s expression recovered to the lower level than the control [Fig. 6(c)]. Injection of CpG-ODN2006 led to induced LvMyD88s expression at all time points [Figs. 6(d)]. Interestingly, LvMyD88 was downregulated at 4 and 8 h post-injection with *V. parahaemolyticus* but later upregulated [Fig. 6(e)]. Lastly, the WSSV infected group showed increased LvMyD88s expression when compared with the control group [Fig. 6(f)].

### LvMyD88 activates *Drosophila* and shrimp AMP promoters

**Figure 7:**
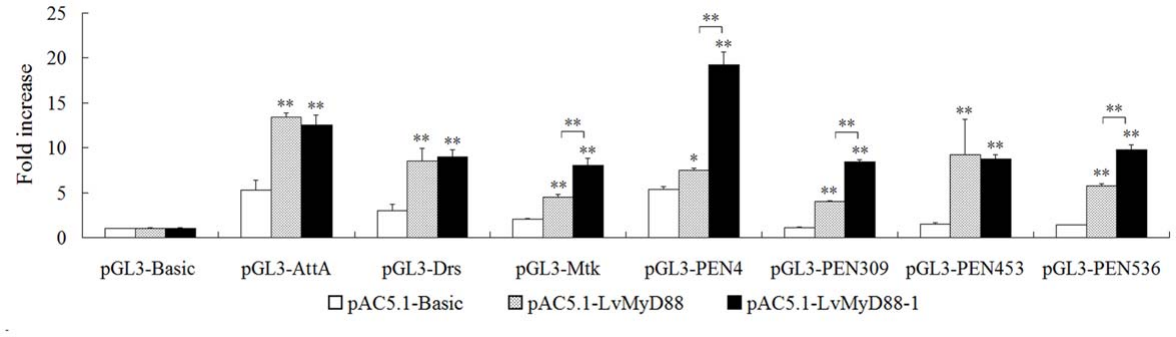
Effects of LvMyD88 and LvMyD88-1 on the promoter activities of *Drosophila* and shrimp antimicrobial peptide genes (AMPs) in *Drosophila* S2 cells. *Drosophila* S2 cells were transfected with the protein expression vector (pAC5.1 empty vector, LvMyD88, or LvMyD88-1), the reporter gene plasmid (pGL3-Basic, pGL3-AttA, pGL3-Drs, pGL3-Mtk, pGL3-PEN4, pGL3-PEN309, pGL3-PEN453, or pGL3-PEN536), and the pRL-TK Renilla luciferase plasmid (as an internal control: Promega, USA). After 48 h, the cells were harvested for measurement of luciferase activity using the dual-luciferase reporter assay system (Promega, USA). All data are representative of three independent experiments. The bars indicate the mean ± SD of the luciferase activity (*n* = 3). The statistical significance was calculated using Student's *t*-test (* indicates *p*<0.05 and ** indicates *p*<0.01compared with control).

In *Drosophila* and shrimp, the AMPs are important immune factors and their expression is believed to be controlled mainly by the NF-κB signal pathway [39], [40], [45], [46]. In shrimp, it has been reported that the NF-κB signal pathway can be activated by Toll, Pelle, TRAF6, Dorsal and Relish [37]–[42]. The present study has demonstrated that both LvMyD88 and LvMyD88-1 were able to activate the promoters of *Drosophila* and shrimp AMP genes. LvMyD88 induced the promoter activities of the *Drosophila* AMPs including AttA (2.54-fold), Drs (2.83-fold), and Mtk (2.23-fold), the *L. vannamei* AMP PEN4 (1.40-fold) and the *P. monodon* AMPs such as PEN309 (3.55-fold), PEN453 (6.22-fold), and PEN536 (4.07-fold). Similar inducible effect was also detected for LvMyD88-1. It must be noted that LvMyD88-1 is more potent to drive the gene promoters of the *Drosophila* Mtk, *L. vannamei* PEN4, and *P. monodon* PEN309 and PEN536 than LvMyD88 (Fig. 7). All these data suggest that LvMyD88 may be involved in regulating NF-κB signaling.

### LvMyD88 activates WSSV gene promoters

**Figure 8:**
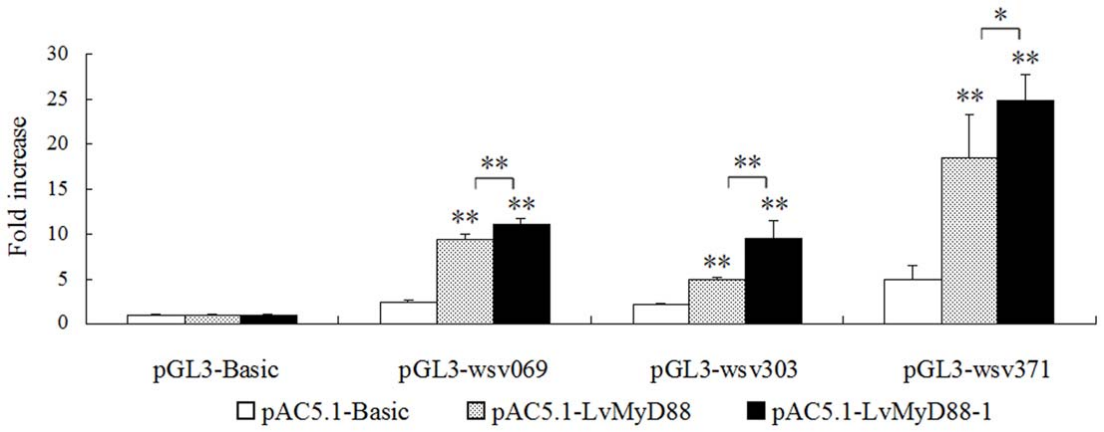
Overexpression of LvMyD88 and LvMyD88-1 activate viral gene expression. The S2 cells were transfected with 0.3 µg of the protein expression vector (pAC5.1 empty vector, LvMyD88, or LvMyD88-1), the reporter gene plasmid (pGL3-Bsic, pGL3-wsv069, pGL3-wsv303, or pGL3-wsv371) and the pRL-TK Renilla luciferase plasmid (as an internal control; Promega, USA). At 48 h after transfection, the cells were harvested and analyzed using the dual-luciferase reporter assay system (Promega, USA). All data are representative of three independent experiments. The bars indicate the mean±SD of the luciferase activity (*n* = 3). The statistical significance was calculated using Student's *t*-test (* indicates *p*<0.05 and ** indicates *p*<0.01compared with the control).

A previous study suggested that the wsv069 (ie1), wsv303, and wsv371 genes are upregulated upon activation of LvPelle and LvDorsal [28]. This study showed both LvMyD88 and LvMyD88-1 upregulate viral gene expression, suggesting potential interaction between viral proteins and the host MyD88/NF-κB pathway. Expression of both LvMyD88s resulted in elevated luciferase activities for all the viral gene promoters (Fig. 8), with LvMyD88-1 gave rise to higher promoter activities than LvMyD88.

## Discussion

In panaeid shrimp, most of the components involved in the Toll pathway of *Drosophila* were identified [37]–[42]. However, the key adaptor protein co-ordinating the Toll pathway, MyD88, remains elusive. In this study, we cloned the MyD88 gene for the first time in panaeid shrimp, and discovered two variants of LvMyD88 (LvMyD88 and LvMyD88-1). Our data showed that both LvMyD88s were capable of inducing the expression of AMP genes in *Drosophila* and shrimp. Furthermore, overexpression of LvMyD88s may also promote viral gene expression.

MyD88 has been identified in both vertebrates and invertebrates. However, MyD88 variants have been found only in humans, mice and chicken [13], [15], [16]. Human and murine MyD88 gene contains five exons and four introns [21]. Alternative splicing of exon 2 leads to a protein lacking the inhibitor of DNA (ID) domain usually found between DD and TIR domains [29], [30]. Additional splicing variant of human MyD88 is *EXC09* which lacks 19 bp in nucleotides 659–677 [13]. In chicken, MyD88 variants were not generated from alternative splicing of MyD88 pre-mRNA [16]. Chicken MyD88-2 had a single nucleotide deletion at position 859, which resulted in a protein with 77 aa residues shorter than MyD88-1, the wild type of MyD88 with 376 amino acids. Chicken MyD88-3 had a 8 aa deletion at the N terminus [18]. In this study, two MyD88 variants have been found for the first time in an invertebrate species *L. vannamei*. Sequences analysis showed that the LvMyD88 conformed to the consensus GT/AG rule for splicing but LvMyD88-1 did not [47]. It is likely that the difference between LvMyD88 and LvMyD88-1 is due to alternative splicing utilizing a non-canonical splice site. However, we do not know how LvMyD88-1 generated according to the present results. Future studies are need.

The two LvMyD88 variants were constitutively expressed in *L. vannamei*. Previous studies showed that MyD88 expression was changed after activation of TLR signaling both in vertebrate and invertebrate. Rock bream MyD88 has been shown to become up-regulated in blood, spleen and head kidney in response to experimental challenge with LPS and *Edwardsiella tarda* [26]. In Japanese flounder peripheral blood leukocytes, MyD88 was also found to be strongly up-regulated in response to experimental exposure to LPS, PGN, and poly I∶C [21]. In scallop, MyD88 was up-regulated in primary cultured hemocytes after LPS and PGN treatment [27]. To better understand the roles of LvMyD88 in response to exposure to various potential pathogens in *L. vannamei*, the expression of LvMyD88 was investigated in hemocytes after stimulation with the ligands of different TLRs, gram-negative bacterium *V. parahaemolyticus*, gram-positive bacterium *S. aureus* and viral pathogen WSSV. After poly I∶C stimulation, the transcriptional level of the LvMyD88 was lower than that of the control group at all time points except 4 h and 12 h. LvMyD88 was up-regulated after stimulation with LPS and CpG-ODN2006, *V. parahaemolyticus*, *S. aureus* and WSSV. Particularly, LvMyD88 were strongly upregulated after LPS and *S. aureus* challenged. It is plausible that the obvious differences in the responses in terms of MyD88 protein levels were probably due to the differences in the TLRs involved in recognizing the different PAMPs involved in each stimulation experiment. All the results suggested that LvMyD88 may play a role in innate immune in *L. vannamei*.

In *Drosophila*, it is known that the Toll pathway is central to host anti-bacterial and anti-viral response by regulating the expression of the immune related genes, including AMPs [48]–[50]. Similarly, in shrimp, AMP genes including Penaeidin, Crustin and antilipopolysaccharide factors (ALFs), are induced after bacterial and viral infection [51], [52]. Like their *Drosophila* counterparts, expression of shrimp AMPs is controlled by the Toll pathway through the conserved transcription factors such as NF-κB, GATA and AP-1 [52], [53]. In this study, LvMyD88 increased the promoter activities of several *Drosophila* and shrimp AMP genes in *Drosophila* S2 cells. Interestingly, LvMyD88 also enhanced the expression of wsv069 (ie1), wsv303, and wsv371, functioning as LvPelle and wsv449 (Fig. 8) [37]. All the results suggested that LvMyD88 may play the role in anti-bacterial and anti-viral response through the Toll pathway.

In summary, this study of LvMyD88 in *L. vannamei* supported the view that an evolutionarily conserved TLR/MyD88/Tube/Pelle/TRAF6/NF-κB signaling pathway may exist in shrimp [37]. It may help better understand the innate immune pathway in shrimp, which would be beneficial to the prevention of various diseases in shrimp culture, and provide valuable information for the study of origin and evolution of innate immunity.
